# Surgery for longer duration supranuclear ophthalmoplegia secondary to brain stem cavernoma: A case report and literature review

**DOI:** 10.1097/MD.0000000000037221

**Published:** 2024-04-05

**Authors:** Wenyan Sheng, Wei Ge, Liwei Zhu

**Affiliations:** a Department of Ophthalmology, Hangzhou Red Cross Hospital (Zhe Jiang Chinese Medicine and Western Medicine Integrated Hospital), Hangzhou, Zhejiang, P.R. China.

**Keywords:** brainstem, case report, cavernoma, gaze palsies, ophthalmoplegia, strabismus, supranuclear

## Abstract

**Background:**

Previous reports revealed that patients with acquired paralytic strabismus caused by central nervous system diseases are primarily affected by the etiology and treatment of the condition. Strabismus correction for these acquired paralytic strabismus should be performed as soon as the primary disease has been stabilized for 6 months in order to archive a favorable surgical outcome.

**Case:**

We followed an infrequent case of longer-lasting supranuclear ophthalmoplegia secondary to brain stem cavernoma.

**Observation:**

A 25-year-old Chinese Han female developed aberrant head posture and ipsilateral conjugate gaze palsies 8 years after the first brainstem hemorrhage caused by pontine cavernoma. The patient was diagnosed with supranuclear ophthalmic palsy and brain stem cavernoma after surgery. A resection–recession procedure along with a rectus muscle transposition was performed. The patient’s abnormal head position disappeared, with a normal primary position.

**Conclusion:**

Resection–recession procedures combined with rectus muscle transposition works very well for longer duration large-angle strabismus caused by brain stem cavernoma.

## 1. Introduction

Approximately 0.4% to 0.8% of the general population have been reported to have cerebral cavernous malformations (CMs), which are vascular defects without interposed brain tissue.^[1]^ Additionally, brain stem CM (BSCM) is a specific type of cavernoma located at the stem, accounting for 9% to 35% of CMs.^[2]^ The disease may occur at any age, frequently in young adults, leading to acute hemorrhage.^[3]^ Nevertheless, BSCM hemorrhage seldom results in severe impairment of extremity movements or consciousness; instead, most manifest as ocular symptoms causing central paralytic strabismus, which is primarily irreversible even after performing hemangioma resections.^[4]^ Menon et al^[5]^ reported 52 BSCM cases, in which 94.2% demonstrated cranial nerve dysfunction, and 48.1% had hemiparesis with no conscious impairment. Chen et al^[3]^ reported cranial nerve dysfunction in 65.8% of BSCM cases. Brain stems cavernoma hemorrhage occurs gradually in lesser amounts and rarely produces significant hematomas that seriously hampers motor and cognitive functions in contrast to cerebral hemorrhage caused by hypertension, arteriovenous malformations, and tumors.^[6]^ The primary form of treatment is lesion resection, while cranial nerve damage is the most typical postoperative side effect.^[7]^

We present a case of longer-lasting supranuclear ophthalmoplegia due to a brain stem cavernoma, which persisted as paralytic strabismus 8 years after the initial brainstem hemorrhage caused by the pontine cavernoma. It is universally acknowledged that, strabismus correction should be performed as soon as the primary disease has been stabilized for 6 to 8 months. Since prolonged disease duration may generate antagonistic muscle contractions, which increase the difficulty of the operation and may affect the efficacy of the surgery. The paralytic strabismus in our patient progressed over a period of up to 8 years, but the optimal repair was achieved thanks to a carefully planned surgical procedure.

## 2. Case presentation

A 25-year-old female patient, who complained of bilateral gaze to the left side for 8 years, was admitted to the Department of Ophthalmology of Hangzhou Red Cross Hospital (Hangzhou, Zhejiang, China) on April 13, 2022.

The Institutional Review Board for the Protection of Human Subjects of Hangzhou Red Cross Hospital approved this study, which adhered to the tenets of the Declaration of Helsinki. The patient signed informed consent before treatment initiation.

**Figure 1.**
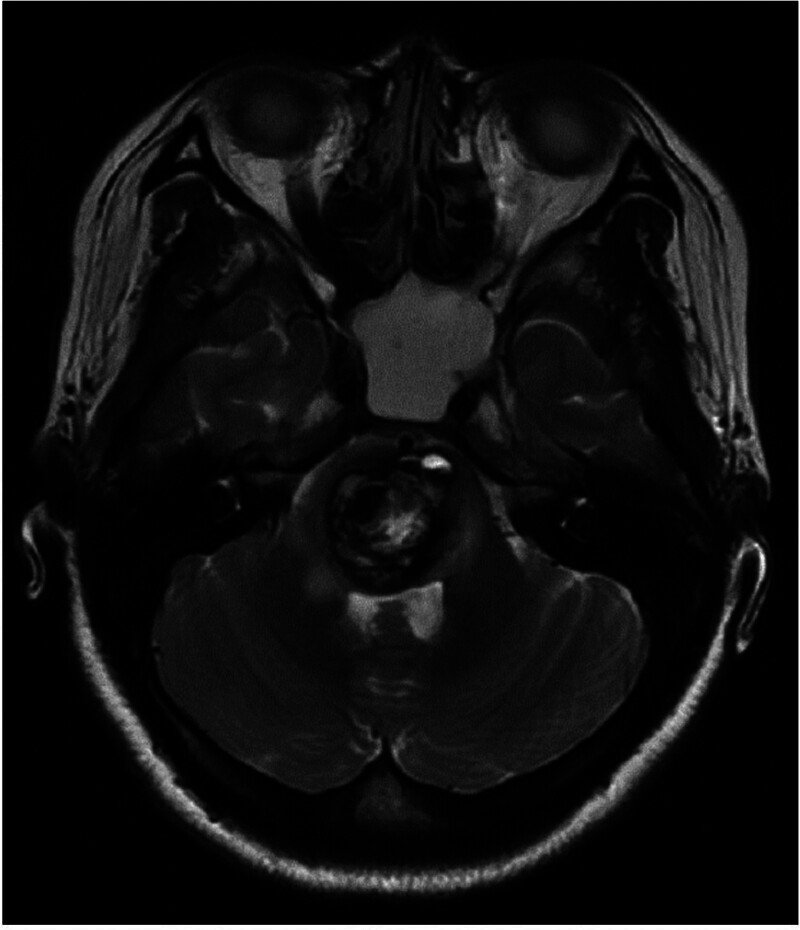
Preoperative brainstem MRI of the patient. Cerebral MRI revealed mass-like confounding of T1 and T2 signals within the pontine brain, SWAN showed hypointense signals, and central plaque hyperintense signals; little edema around lesion (SWAN: T2* weighted angiography). MRI = magnetic resonance imaging.

**Figure 2.**
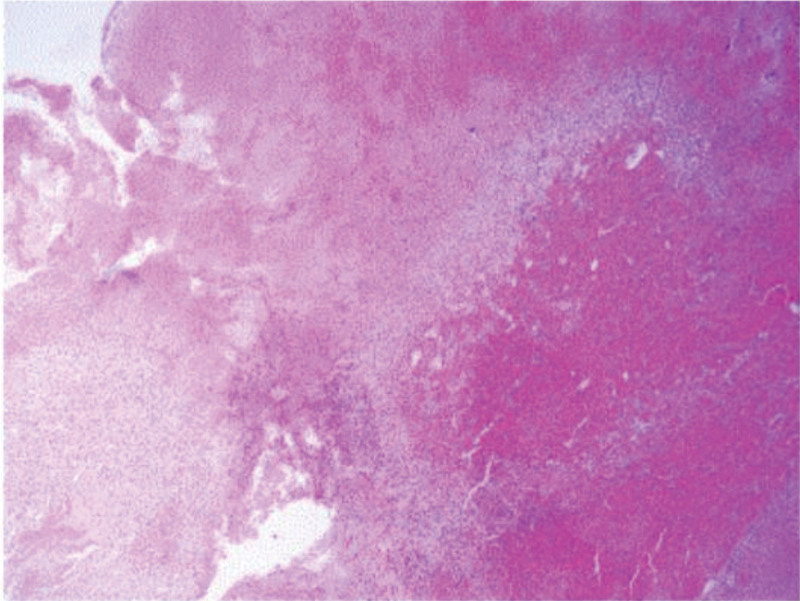
Postoperative pathologic section diagnosis of cavernous hemangioma.

The patient complained of a sudden headache, left extremity paralysis, and palsies of ipsilateral conjugate gaze in 2014. She was diagnosed with “stem-brain hemorrhage” and progressively achieved symptomatic relief after conservative treatments. Similar symptoms reappeared on November 29, 2017, and “pontine cavernoma with hemorrhage” was diagnosed in another hospital. She underwent a “brainstem cavernoma dissection” in neurosurgery department. Figure 1 shows preoperative brainstem magnetic resonance imaging results. During the operation, an ancient hematoma containing a morula-like vessel mass of 0.5 × 0.5 × 0.5 cm was noticed in the pons. Figure 2 shows postoperative pathology reports.

The patient had no history of hypertension, diabetes, or any other ophthalmic diseases. No family members had similar diseases.

**Figure 3.**
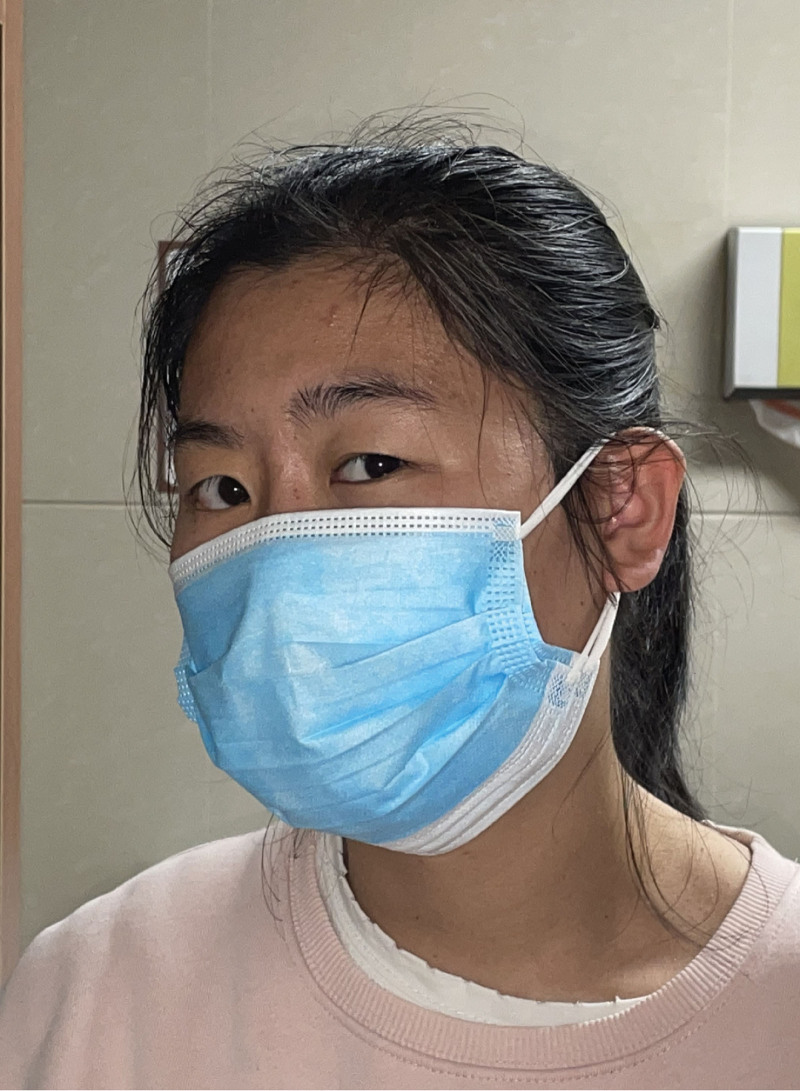
Preoperative photographs of the 28-y-old female patient with ipsilateral gaze palsy. The patient turned her head to the left side in order to get binocular single vision in front.

**Figure 4.**
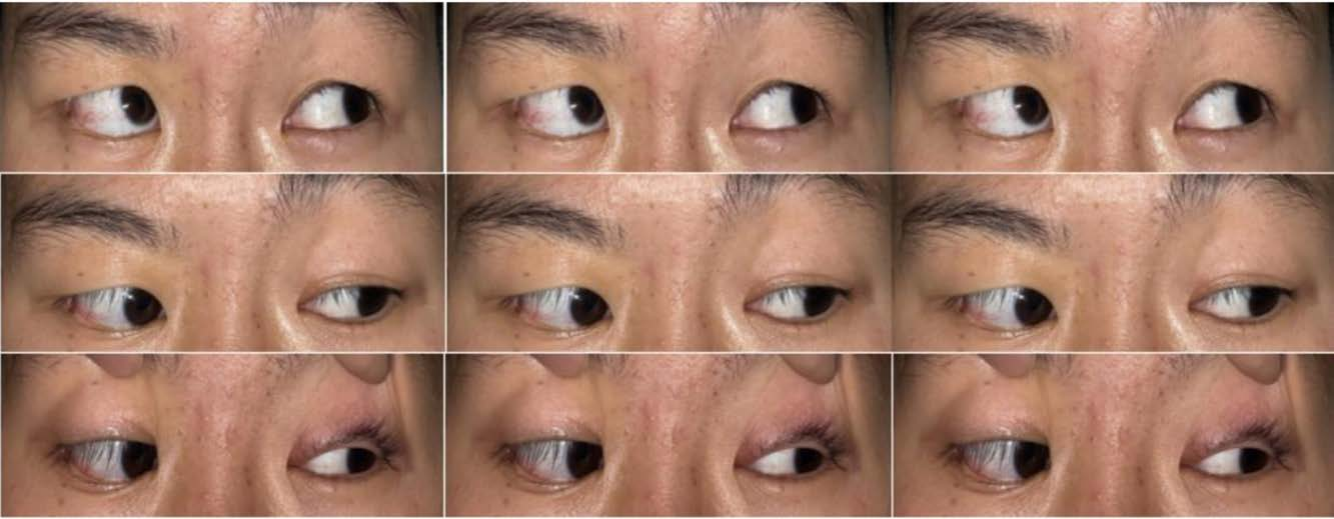
Preoperative photograph in 9 eye positions of the patient. The patient had total palsy in the right lateral rectus muscle and the left medial rectus muscle.

The patient’s best-corrected visual acuities during the physical exam were 4.9 and 5.0 in the right and left eyes, respectively. Figure 3 illustrates the preoperative ocular position. Her head was turned to the right, and her eyes were turned to the left. Both eyes could not adduct (Fig. 4). Bilateral anterior segments under a slit-lamp microscope were examined but revealed no abnormalities. The pupils had no relative afferent pupillary defects. The discs in both eyes appeared normal during the fundus examination. Additionally, right-sided peripheral facial paralysis with a broader right eyelid fissure, partial right eye closure, shallowness of the right nasolabial groove, and suitable forehead wrinkle was observed.

Our patient had peripheral facial paralysis on the right side, left extremities paralysis, and palsies of the ipsilateral conjugate gaze when the stem-brain hemorrhage started. The lesion was primarily seen on the right side of the pons, as confirmed by preoperative magnetic resonance imaging and intraoperative findings, and the postoperative pathologic diagnosis revealed cavernoma. The diagnosis of cavernous hemangioma of the pons after excision and supranuclear ophthalmic muscle palsy was established. It is not an easy task for ophthalmologists to make a clear diagnosis of intercranial lesions. The diagnosis and treatment results of the patient’s previous neurosurgery have provided us with a clear diagnostic basis.

Our procedure was conducted on this patient in 2 stages. The right medial and lateral rectus muscles experienced recessions of 10 mm during the initial process. The patient underwent a second operation after 4 weeks, during which the right lateral rectus muscle was shortened by 10 mm and the left medial rectus muscle was shrunk by 8 mm. Additionally, the right eye’s inferior and superior rectus muscles were cut in half, and their insertions were stitched to the insertion of lateral rectus muscle.

**Figure 5.**
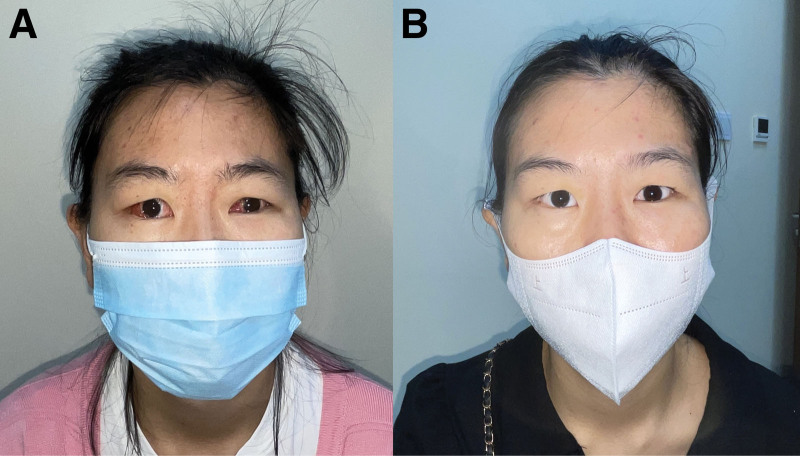
Postoperative photographs of the patient. The patient’s abnormal head position disappeared with normal primary position. (A) One week after the second operation. (B) Six months after the second operation.

**Figure 6.**
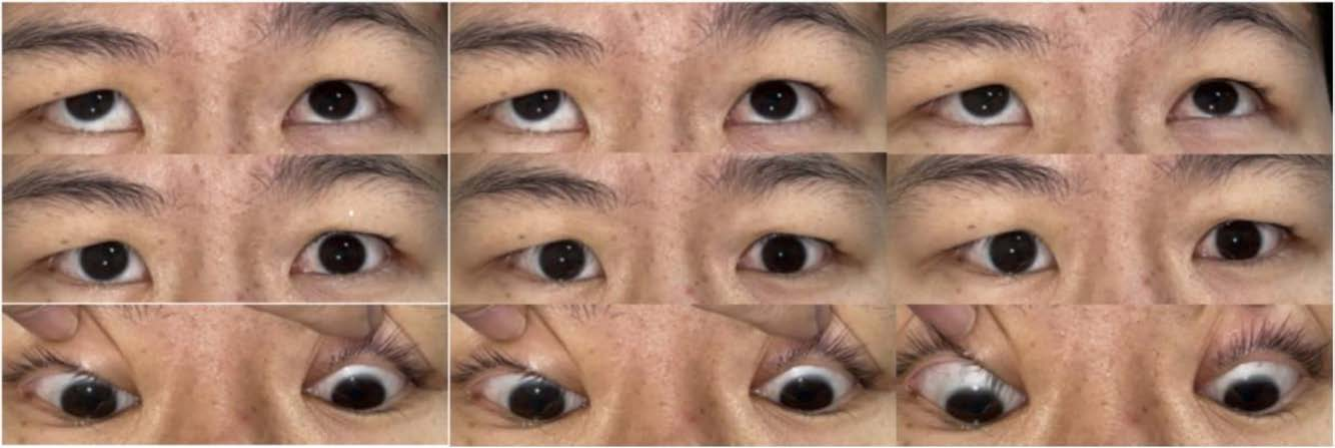
Postoperative photographs in 9 eye positions of the patient. The patient got corrected ocular alignment in the primary position.

The compensated head position disappeared after the second operation, as shown in Figure 5. The eyes were in corrected ocular alignment in the primary position (Fig. 6), without any signs of anterior segment ischemia, at the postoperative 6-month follow-up. The patient was quite satisfied with the result.

## 3. Discussion

The ocular motor system, including the cerebellum, extraocular muscles, vestibular structure, and ocular motor nerves and brainstem nuclei originating in the cerebral cortex, controls eye movements. Central ophthalmoplegia is divided into supranuclear, internuclear, nuclear, and infranuclear levels based on anatomic location. The pursuit, saccadic, vestibular, and vergence ocular motor subsystems can be specifically affected at the supranuclear level. Internuclear ophthalmoplegia is a disorder of the internuclear gaze pathways, caused by the medial longitudinal fasciculus lesion. The affected eye in internuclear ophthalmoplegia cannot adduct during lateral gaze, but it can adduct normally during convergence. Nuclear lesions differ from lesions of the corresponding cranial nerve since the motoneurons for the individual eye muscles may be specifically grouped. Infranuclear lesions affect the fasciculi of the third, fourth, or sixth nerves before they leave the brain stem and cause symmetrical eye movement disorder as a peripheral lesion.^[8,9]^

The inability of the eyes to move conjugately in a specific direction is referred to as gaze palsy. Horizontal gaze palsy is caused by a lesion in the ipsilateral side of the pons, the frontopontine pathways, or the contralateral hemisphere.^[9]^ Locating the craniel lesion accurately may be a difficult task for most of ophthalmologists. Our patient had a pontine lesion that mostly affected the right side and resulted in permanently saccadic gaze palsy on the ipsilateral side of the lesion. A pontine cavernoma in our patient repeatedly bled, causing a bigger lesion. She also experienced contralateral hemiparesis caused by damage to the nearby pyramidal tract and ipsilateral peripheral facial paralysis brought on by adjacent facial nerve fascicle injury.

Complete paralytic strabismus brought on by severe brain nerve damage is characterized by a significant angle of deviation and a notable motility restriction without the capacity to reach the mid-line. As we know, if the primary disease is stable, the patient would wait for 6 to 8 months for recovery. After 6 to 8 months’ recovery, the paralytic strabismus should be treated as soon as feasible because secondary alterations, such as antagonistic muscle contracture, will make the procedure more difficult and may reduce its effectiveness. Surgery should be considered after trying conservative measures, such as botulinum toxin A injections and Fresnel prisms.^[10,11]^ These treatments are distinct from conventional surgical techniques for comitant strabismus and do not follow a set procedure because of the intricacy of the condition and the difficulties in predicting the surgical outcome. It is more challenging with the surgical design. Surgery in these patients mainly aimed at enhance ocular mobility and attractive appearance, get rid of diplopia, and broaden the binocular visual field.

Large-angle deviations and head twists caused by various types of paralytic strabismus can be surgically treated in several ways. Simple resection–recession techniques cannot sufficiently increase the range of binocular single vision when the impaired muscle has little or no contractile force.^[12]^ Early in the 20th century, Hummelsheim wrote about a partial rectus muscle transportation method for treating sixth nerve palsy. In this method, the superior and inferior rectus muscles were divided in half and the lateral portion was transposed to the insertion site of the palsy-affected muscle.^[13]^ The risk of anterior segment ischemia can be minimized by carefully preserving the ciliary blood vessels during muscle separation. Generally, partial rectus muscle transportation and its variants are effective in treating paralytic strabismus. Muscle transplantation combined with recession and resection of the medial or lateral rectus muscle is highly effective for big-angle esotropia (>70∆), significant exotropia, and abducens nerve palsy with good abduction.^[14]^ The long-term effects of muscle transplantation in 22 patients were documented by Jethani. Esotropia of >85∆ was present in all patients, and the average follow-up time was 2 years.^[15]^ Arfeen et al^[16]^ compared the Hummelsheim and Jensen techniques when treating persistent sixth nerve palsy. No difference was found in the success rates of the Hummelsheim and Jensen treatments in patients with chronic sixth nerve palsy. Results of increased Hummelsheim and X-type transpositions for the treatment of large-angle strabismus are reported by Gokoffski et al.^[12]^ Enhanced Hummelsheim transposition techniques are effective for paralytic strabismus with isotropic deviations while X-type transpositions are successful for exotropic deviations resulting from significant inferior rectus injury.

Our patient had lateral gaze palsy with large-angle deviations for up to 8 years, regular supra-maximal recession–resection procedure was insufficient. The paralyzed muscles in our patient, were fully weak and had contracted antagonistic muscles. So we selected the resection–recession techniques combined with rectus muscle transposition, which effectively correct ocular alignment in the primary position, and eliminate the compensatory head position. During the follow-up for the next 6 months, the patient’s eye position remained good and there were no complications such as anterior segment ischemia.

## 4. Conclusion

The varied etiologies and complex clinical presentation of central ophthalmoplegia make the diagnosis difficult. The degree of extraocular muscle dysfunction and the ocular motility deficit should be carefully assessed to design an operation that would yield the greatest benefit.

## Acknowledgments

Thanks to Enago (www.enago.cn) for Language embellishment. The authors thank to the guardians and relatives of the patient for conceding their consent for the case report.

## Author contributions

Funding acquisition, investigation: Wei Ge.

Supervision: Liwei Zhu.

Writing – original draft: Wenyan Sheng.

Writing – review & editing: Wenyan Sheng, Liwei Zhu.
